# Nonlocal phase-change metaoptics for reconfigurable nonvolatile image processing

**DOI:** 10.1038/s41377-025-01841-x

**Published:** 2025-05-06

**Authors:** Guoce Yang, Mengyun Wang, June Sang Lee, Nikolaos Farmakidis, Joe Shields, Carlota Ruiz de Galarreta, Stuart Kendall, Jacopo Bertolotti, Andriy Moskalenko, Kairan Huang, Andrea Alù, C. David Wright, Harish Bhaskaran

**Affiliations:** 1 Department of Materials, University of Oxford, Parks Road, Oxford, OX1 3PH UK; 2 Centre for Metamaterial Research and Innovation, University of Exeter, Exeter, EX4 4QF UK; 3 Photonics Initiative, Advanced Science Research Center, City University of New York, New York, NY USA; 4 Physics Program, Graduate Center, City University of New York, New York, NY USA

**Keywords:** Optical materials and structures, Other photonics

## Abstract

The next generation of smart imaging and vision systems will require compact and tunable optical computing hardware to perform high-speed and low-power image processing. These requirements are driving the development of computing metasurfaces to realize efficient front-end analog optical pre-processors, especially for edge detection capability. Yet, there is still a lack of reconfigurable or programmable schemes, which may drastically enhance the impact of these devices at the system level. Here, we propose and experimentally demonstrate a reconfigurable flat optical image processor using low-loss phase-change nonlocal metasurfaces. The metasurface is configured to realize different transfer functions in spatial frequency space, when transitioning the phase-change material between its amorphous and crystalline phases. This enables edge detection and bright field imaging modes on the same device. The metasurface is compatible with a large numerical aperture of ~0.5, making it suitable for high resolution coherent optical imaging microscopy. The concept of phase-change reconfigurable nonlocal metasurfaces may enable emerging applications of artificial intelligence-assisted imaging and vision devices with switchable multitasking.

## Introduction

In the past decade, convolution neural networks (CNNs), a class of deep learning models for artificial intelligence (AI), have been widely used to solve target classification and recognition problems, with applications ranging from image-based medical diagnostics to real-time object detection for self-driving vehicles^1–4^. Practically, convolution layers—the fundamental front-end building blocks in CNNs—are responsible for most of the energy consumption, mainly due to the numerous multiplication operations they need to perform, which also ultimately slows down the processing speed in conventional electronic processors. Using light as carriers to process information has been a long-term vision due to the lower latency, smaller loss, and larger data throughput. Problems like relatively large footprints and scale-up issues make full optical solutions challenging but part substitution by photonic devices at the front end of a system is a feasible way to push the boundary of this field. There has been substantial progress in developing photonic integrated circuits^5,6^, while alternatively free-space optics can manipulate input coherent light where two-dimensional information such as images is encoded directly, and process information without costly pre- and post-processing such as data vectorizations and electro-optical conversions. By operating directly in the spatial frequency space, these devices can also dramatically speed up computation and reduce energy consumption compared to digital matrix multipliers.

A traditional optical method to process information in the spatial frequency space is to use a 4-f system^7^, in which a lens transforms the spatial frequency space to the real space on the confocal plane and a spatially varying optical filter with position dependent transmittance is applied on that plane. Recently, metasurfaces have emerged as promising platforms in image processing. One class of metasurfaces is “local” metasurfaces^8–14^ that tailor the spatial wavefront by engineering the aperture response as a function of the spatial position. Directly using this as a spatially varying filter in a 4-f system is one option to implement spatial frequency modulation and image processing^15^ but the system will be bulky due to the long 4-f distance and limited by paraxial approximation. Engineering the point spread function of a metalens—a main class of local metasurfaces—is another way to implement spatial frequency modulation indirectly by doing convolution in real space directly without a 4-f system^16–18^, but it relies on precise optimization and control of spatially varied nanostructures’ geometry. In contrast, a “nonlocal” metasurface^19–26^, whose meta-atoms tailor the input wave collectively and result in real space invariant but spatial frequency space variant response, can directly modulate the Fourier component of light fields and can therefore be readily integrated into the front- and/or back-end of an optical system. This in turn leads to compact designs that can be easily incorporated into a range of optical systems. Moreover, unlike local metasurfaces, nonlocal metasurfaces possess geometrically uniform nanostructures, so are more compatible with facile and large-area nanofabrication approaches, such as interference photolithography and nanoimprint lithography. Due to these advantages, nonlocal metasurfaces are emerging as promising platforms to process information in the optical domain, with interesting applications such as image differentiators^27–31^, space squeezers^32–34^, light bullet generations^35^ and reciprocal lenses^36^ recently reported in the literature. However, such approaches so far have invariably used static metasurfaces that can perform a single function or processing task, limiting their impact and appeal for complex systems. Integrating multiple functionalities over a single nonlocal metasurface may bring significant processing advantages. In turn, this requires a nonlocal metasurface with capability for reconfiguration, which is the focus of the present work.

Very different from traditional local metasurfaces where the phase and amplitude response depend on meta-atoms locally and require individual control of meta-atoms, nonlocal metasurfaces have spatially uniform optical response, which makes globally switching metasurfaces enough to implement various functions. A reconfigurable nonlocal metasurface capable of image processing was recently proposed and demonstrated on a mechanical stretchable polydimethylsiloxane (PDMS) based platform^37,38^. Alternatively, phase-change materials (PCMs), the approach we use here, are good candidates to be applied for reconfigurable metasurfaces, due to their relatively large refractive index difference (Δ*n* > 0.7) between their crystalline and amorphous states, and the non-volatility of such states, i.e., no energy consumption is required to remain in any particular state, although the switching operation itself consumes energy. Only recently have these materials been used to introduce reconfigurability in metasurfaces, such as focus-varied metalens^39,40^, beam steering devices^41,42^, filtering^43^ and more, raising significant interest in the area. The recent discovery of low-loss and lossless PCMs^44^ and the substantial progress in developing integrated heaters capable of in-situ switching of PCM layers and meta-atoms^41,45–48^, make them suitable for all-solid-state integrated meta-devices.

In this work, we theoretically propose and experimentally demonstrate a reconfigurable nonlocal metasurface made of Sb_2_Se_3_, an optical lossless PCM in the near-infrared region. This phase-change nonlocal metasurface showcases a different transfer function in the spatial frequency space when switched between its amorphous and crystalline phases, and, in the example demonstrated here, is able to perform edge detection and bright field imaging in the two distinct PCM phases. Our nonlocal metasurface is compatible with a numerical aperture (NA) of ~0.5, and an optical bandwidth of 50 nm corresponding to fractional bandwidth of 1/21 in theory, which enables applications in coherent optical imaging systems with high optical resolution. Furthermore, a dual-functionality coverslip based on the fabricated phase-change nonlocal metasurface (meta-coverslip) is demonstrated. The introduced concept of phase-change nonlocal metasurfaces may also enable all-solid-state dynamically tunable light modulators operating in the spatial frequency space.

## Results

**Fig. 1:**
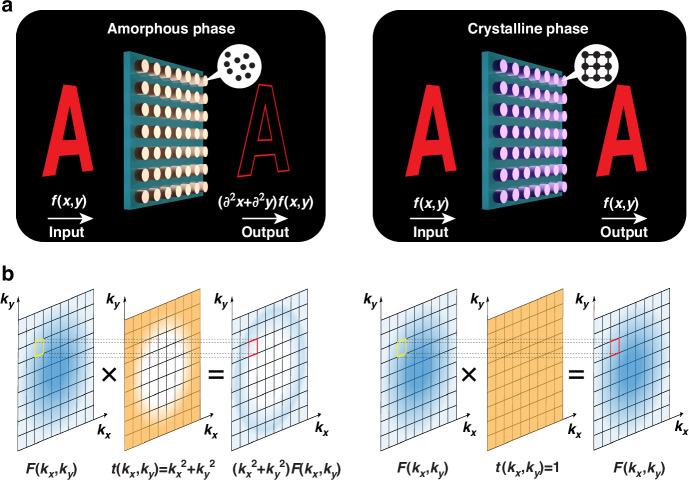
Dual function phase-change nonlocal metasurface. **a** 3D illustration of the phase-change nonlocal metasurface performing the dual functions of edge detection in the amorphous phase and bright field imaging in the crystalline phase of Sb_2_Se_3_. **b** Schematic of the working principle of the metasurface in k-space, corresponding to the Fourier transform of the operation in real space

**Fig. 2:**
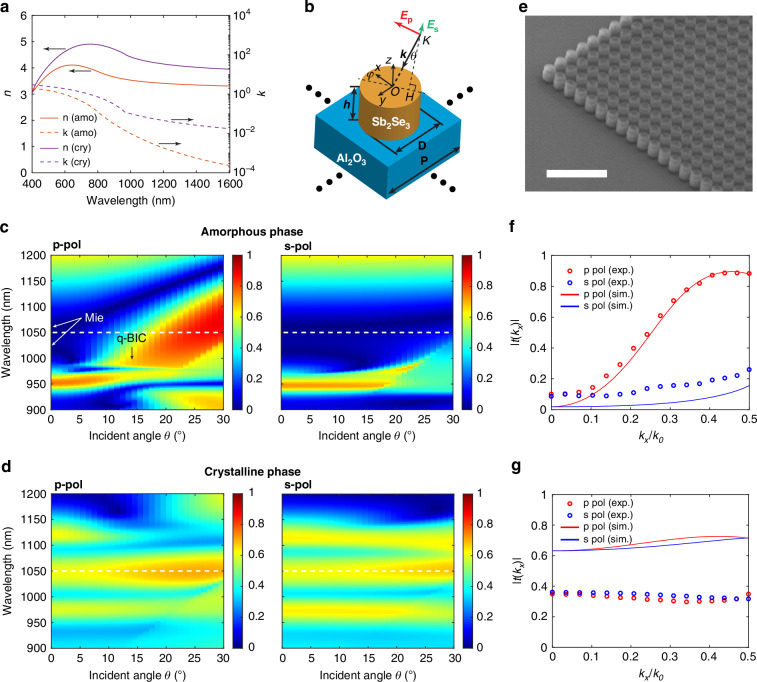
Optical transmission properties of the nonlocal metasurface varying the Sb_2_Se_3_ phase. **a** Measured refractive index of Sb_2_Se_3_ in the amorphous and crystalline phase. **b** 3D model of a unit cell of the metasurface with the configuration of incident light. *h* = 300 nm, *D* = 350 nm, and *P* = 460 nm. **c**, **d** Simulated magnitude of transmissive coefficient spectra vs. incidence angle for the metasurface in the amorphous and the crystalline phase, where the azimuthal angle φ = 0°. **e** Representative SEM image of a fabricated metasurface. Scale bar: 2 μm. **f**, **g** Calculated and measured spatial frequency dependent transfer function of the metasurface in the amorphous (**f**) and crystalline phase (**g**) at the working wavelength of 1050 nm, corresponding to the dashed lines in (**c**) and (**d**)

A three-dimensional (3D) schematic of our conceptual phase-change nonlocal metasurface is shown in Fig. 1a. The metasurface consists of arrayed PCM nanopillars on a transparent substrate. With the nanopillars in different phase-states (amorphous or crystalline), the metasurface performs different functions. Here, the input image’s edge is retrieved after light from the original image passes through the metasurface when the PCM is in the amorphous phase, i.e., edge detection mode, while no transformation happens when the PCM is in its crystalline phase, producing a bright field mode image. In this work, the nanopillars are realized by depositing and etching optically low-loss PCM Sb_2_Se_3_ on a sapphire substrate. The edge detection is implemented via a Laplacian operator in real space, which corresponds to a multiplication operator with a parabolic form in spatial frequency space (or called k-space below), while the bright field mode corresponds to a pass-through-type transformation (shown in Fig. 2b). Therefore, in order to implement a dual function nonlocal metasurface, the transfer function *t*(*k*_*x*_, *k*_*y*_) in k-space should be proportional to *k*_*x*_^2^+*k*_*y*_^2^ in the edge detection mode, while remaining flat (*t*(*k*_*x*_, *k*_*y*_)=1) in the bright field mode^24^. Our general method is to first design the metasurface working in one material phase in the edge detection mode which relies on a vertically oriented dipole resonance^24,28^ and associated quasi bound states in the continuum (BICs)^49–51^ to create a transmittance strongly dependent on incident angle, then to check whether transmittance is independent of incident angle in the other phase, so yielding the bright field mode. This requires the dipole resonance responsible for the incident angle dependent transmittance in the amorphous phase to shift far enough after the material crystallization such that the resonant bandwidth is fully off the working wavelength and the dependence on incident angle disappears. The large refractive index change of PCMs between the two phases makes the large resonance shift possible and helps to achieve the dual function image processing we desire.

Before optimizing the spatial frequency dependent (i.e. k-dependent) transmittance, we measured the complex refractive index of the amorphous and crystalline Sb_2_Se_3_ film using an ellipsometer. The measured results, shown in Fig. 2a, demonstrate that Sb_2_Se_3_ has low optical loss (imaginary part of the refractive index <0.1) at wavelengths above 1000 nm in either phase, and the change of the real part of the refractive index between the two phases can be as large as 0.7 to 1 in the near-infrared region. In turn, the resonant wavelengths of the nanopillars can shift significantly between the two phases, resulting in a large contrast of the transfer function, which gives opportunities to implement different functions on a device with the fixed geometry.

We first design the metasurface working for edge detection in the amorphous phase and then check if the transfer function in the crystalline phase satisfies the bright field mode. The 3D model of the nanopillar unit cell with the geometric parameters and the illumination configuration are shown in Fig. 2b. We simulated the transmissive coefficient spectra of a periodic array of Sb_2_Se_3_ nanopillars—with sizes and periodicity specifically optimized to deliver edge detection in the amorphous state and bright field imaging in the crystalline state—as a function of incident angle with varied incident polarization states and structural phases of Sb_2_Se_3_. Here the transmissive coefficient is defined by the square root of the diffractive efficiency of the 0^th^ transmissive order. The diffraction efficiency of the metasurface was simulated by the rigorous coupled wave analysis (RCWA) method^52^. The simulated spectra exhibit a strong angle dependent contrast in the p-polarized incident state in the amorphous phase (Fig. 2c), which can be explained by the synergy of multiple resonances. The in-plane electric and magnetic dipole Mie resonances of the disks are close to each other in the spectrum at normal incidence, creating a broad band with almost zero transmittance from 980 nm to 1150 nm at normal incidence. With the increase of incidence angle, the Mie resonance induced spectral band is squeezed by the emergence of a nonlocal out-of-plane resonant mode. This mode is a symmetry-protected BIC, fully decoupled from free space at the Γ point, so it cannot be excited by normal incident light. At oblique incidence the symmetry is broken and the radiative decay significantly increases as the wave vector moves away from the Γ point, which leads to a quasi-BIC (q-BIC) mode coupled to the p-polarized oblique incident light, causing more transmission for high spatial frequencies. A detailed BIC mode analysis is shown in Supplementary Information (Note A). By contrast, there is not much change in the spectra with the increased incident angle in the crystalline phase, as shown in Fig. 2d. No significant change of transmittance as a function of the incident angle was observed in the s-polarization state for either phase of the material. From the simulations shown in Fig. 2c, d, the metasurface behaves as a high-pass filter in spatial frequency in the amorphous phase and works as an all-pass filter in the crystalline phase from 1030 to 1080 nm. Therefore, our dual function metasurface has 50 nm bandwidth in theory.

We fabricated the metasurface using conventional processes, including Sb_2_Se_3_ film sputtering, electron beam lithography (EBL), and reactive ion etching (RIE) (see *Methods*). The fabricated sample was finally characterized by scanning electron microscopy (SEM) and a representative captured image shows the high quality of the fabrication (Fig. 2e). To validate our design, we used a laser diode with emission wavelength of 1050 nm to measure the incident angle dependent the transfer function (Fig. 2f, g). The transfer function is defined as the magnitude of transmissive coefficient, i.e. square root of the intensity transmittance which can be directly measured. The measurement setup is described in *Methods*. A strong spatial frequency dependent transfer function was experimentally demonstrated for the amorphous phase and p-polarized incidence, which coincides well with the simulation results. The amplitude of the transfer function reaches 0.9 when *k*_*x*_/*k*_0_ approaches 0.5, which means that the edge detection mode is efficiently (81% = 0.9^2^) compatible with an NA of 0.5. In the other cases (crystalline phase or s-polarization), the measured transfer function is not sensitive to the variation of the spatial frequency. The measured transmissive coefficient is a little higher than the simulated results in the amorphous phase for low transmittance. This could be attributed to the imperfect sample fabrication such as geometry uniformity and stitching errors inducing unwanted forward scattering but collected by the detector. This overestimation is further amplified on the plot by the square root when the transmittance is low. In the crystalline phase, the transfer function is flat as a function of spatial frequency, allowing us to image objects in bright field mode. There is an amplitude difference in the transmissive coefficient between measurements (0.35) and simulations (0.65) in the crystalline phase. We attribute this to scattering loss resulting from the increased non-uniformity/roughness of the crystallized metasurface, as commonly observed for phase-change films and shown for our case in Fig. S2. Additional characterization before and after crystallization can be found in Figs. S3, S4. Also see simulated transfer functions of diameter varied uniform metasurfaces in Fig. S5 and simulated transfer functions of disordered metasurfaces with the consideration of random profile changes in Fig. S6. At the technical level, it is possible to constrain the volume change and improve the uniformity by capping with a suitable dielectric layer around the nanopillar.

**Fig. 3:**
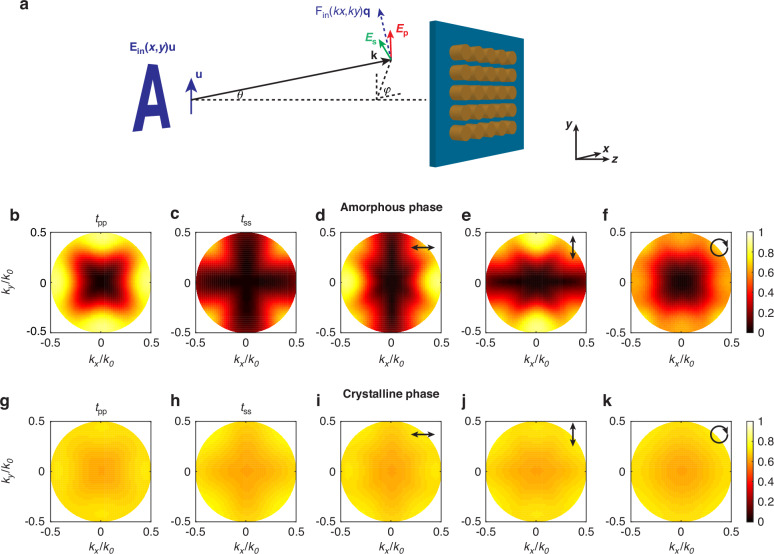
Calculated 2D transfer functions of the metasurface in the k-space. **a** Schematic of relationship between the polarization states of the imaged object and the incident light on the metasurface. **b**, **c** The calculated 2D co-polarization transmissive coefficients for p- and s-polarized incident light relative to the metasurface plane in the amorphouse phase. **d**–**f** The calculated 2D transfer functions for different polarized illuminations on the samples: *x*-polarized, *y*-polarized, and circular polarized. The results of left and right hand circular polarized states are the same due to the symmetry of the metasurface. **g**–**k** The calculated 2D transmissive coefficients and transfer functions of the same configurations as in (**b**–**f**) but in the crystalline phase

We numerically analyze the two-dimensional (2D) transfer function of our designed metasurface used for 2D image processing. We link the input electric field of the imaged object to the s- and p- polarized electric field components relative to the ground plane of the metasurface, and subsequently obtain the transfer function by introducing the simulated transmissive coefficients for s- and p- polarized incidence. The configuration of the input object and the metasurface is schematically shown in Fig. 3a. Starting from the linear polarized light, we denote *E*_in_(*x*,*y*)**u** as the input electric field of the imaged object in real space, where **u** is the unit vector along the polarization direction. The input electric field in k-space is denoted by *F*_in_(*k*_*x*_,*k*_*y*_)**q**(*k*_*x*_,*k*_*y*_), where *F*_in_(*k*_*x*_,*k*_*y*_) is the Fourier transform of *E*_in_(*x*,*y*) and **q**(*k*_*x*_,*k*_*y*_)=(**k**×**u**)×**k/**|**k**|^2^. Then we have the p- and s- polarized components: *E*_p_(*k*_*x*_,*k*_*y*_)=*F*_in_(*k*_*x*_,*k*_*y*_)**q**(*k*_*x*_,*k*_*y*_)∙**e**_p_ and *E*_s_(*k*_*x*_,*k*_*y*_)=*F*_in_(*k*_*x*_,*k*_*y*_)**q**(*k*_*x*_,*k*_*y*_)∙**e**_s_, where **e**_s_ = (**k**×**z**)**/**|**k**| and **e**_p_ =(**k**×**e**_s_)/|**k**| when **k** is not parallel with **z**. For the special case of **k**//**z**, i.e. *k*_*x*_=*k*_*y*_ = 0, we set **e**_s_ = **0** and **e**_p_ = **y** (**y** and **z** are the unit vectors along the *y* and *z* direction, respectively). Hence, the output electric field **F**_out_(*k*_*x*_,*k*_*y*_) after the metasurface in the k-space can be generally written as^31^

1 $${{\bf{F}}}_{{\rm{out}}}({k}_{x},{k}_{y})={F}_{{\rm{in}}}({k}_{x},{k}_{y}){\left(\begin{array}{c}{{\bf{e}}}_{{\rm{p}}}\\ {{\bf{e}}}_{{\rm{s}}}\end{array}\right)}^{{\rm{T}}}\left(\begin{array}{cc}{t}_{{\rm{pp}}}({k}_{x},{k}_{y}) & {t}_{{\rm{ps}}}({k}_{x},{k}_{y})\\ {t}_{{\rm{sp}}}({k}_{x},{k}_{y}) & {t}_{{\rm{ss}}}({k}_{x},{k}_{y})\end{array}\right)\left(\begin{array}{c}{\bf{q}}\cdot {{\bf{e}}}_{{\rm{p}}}\\ {\bf{q}}\cdot {{\bf{e}}}_{{\rm{s}}}\end{array}\right)$$

where the subscript p and s represents the p- and s-polarization, respectively. *t*_pp_, *t*_ps_, *t*_sp_ and *t*_ss_ are the transmissive coefficients of the metasurface from one incident polarization to another output polarization. For example, *t*_ps_ is the transmissive coefficient between the s-polarized incident light and the p-polarized transmitted light. The calculated amplitude of transmissive coefficients as the function of *k*_*x*_ and *k*_*y*_ in different phases are shown in Fig. 3b, c, g, h. In the amorphous phase, the amplitude of the co-polarization coefficient *t*_pp_ significantly increases with the incident angle for all azimuthal angles and we could approximate it as a paraboloid described by *k*_*x*_^2^+*k*_*y*_^2^ in k-space ignoring the slight azimuthal anisotropy. The maxima were reached at the boundary of the k-space circle with values no less than 0.7. In contrast, *t*_ss_ is much lower than *t*_pp_ within the circle of NA < 0.5 and goes to zero when φ approaches 0° and 90°, so we approximately treated it as zero. In the crystalline phase, *t*_pp_ and *t*_ss_ are nonzero and nearly invariant in k-space. The cross-polarization coefficients *t*_ps_ and *t*_sp_ (the amplitude of ~0.2 corresponds to power transmittance of only 4%) are small enough compared with co-polarization coefficients *t*_pp_ and *t*_ss_ (the amplitude of ~0.9 corresponds to power transmittance of 81%) (Fig. S7). Even though our sample was fabricated on sapphire substrate (c-cut), the optical axis is perpendicular to the ground plane, so the tiny birefringence effect of the substrate has no contribution to *t*_ps_ and *t*_sp_. Besides, the very slight anisotropy of the refractive index has negligible effect on the transmission profile in k-space (Fig. S8). From the above analysis we simplify the polarization dependent transmissive coefficient matrix as

2 $$\left(\begin{array}{cc}{t}_{{\rm{pp}}}({k}_{x},{k}_{y}) & {t}_{{\rm{sp}}}({k}_{x},{k}_{y})\\ {t}_{{\rm{ps}}}({k}_{x},{k}_{y}) & {t}_{{\rm{ss}}}({k}_{x},{k}_{y})\end{array}\right)\propto \left\{\begin{array}{c}\left(\begin{array}{cc}{k}_{x}^{2}+{k}_{y}^{2} & 0\\ 0 & 0\end{array}\right),{\rm{amorphous}}\\ \left(\begin{array}{cc}1 & 0\\ 0 & 1\end{array}\right),{\rm{crystalline}}\end{array}\right.$$

This equation implies that our metasurface nearly blocks the s-polarized propagation and applies the Laplacian operator on p-polarized light. Furthermore, to understand the operation by the metasurface in image processing we define the 2D transfer function of the metasurface as

3 $$t({k}_{x},{k}_{y})=\left|{\left(\begin{array}{c}{{\bf{e}}}_{{\rm{p}}}\\ {{\bf{e}}}_{{\rm{s}}}\end{array}\right)}^{{\rm{T}}}\left(\begin{array}{cc}{t}_{{\rm{pp}}}({k}_{x},{k}_{y}) & {t}_{{\rm{ps}}}({k}_{x},{k}_{y})\\ {t}_{{\rm{sp}}}({k}_{x},{k}_{y}) & {t}_{{\rm{ss}}}({k}_{x},{k}_{y})\end{array}\right)\left(\begin{array}{c}{\bf{q}}\cdot {{\bf{e}}}_{{\rm{p}}}\\ {\bf{q}}\cdot {{\bf{e}}}_{{\rm{s}}}\end{array}\right)\right|$$

This allows us to calculate the 2D transfer function of the imaged object illuminated by different polarizations of light via numerically simulated polarization dependent transmissive coefficients. The calculated results for *x*-polarized, *y*-polarized and circular polarized incidence in the amorphous phase are shown in Fig. 3d–f. The transfer function is close to *k*_*x*_^2^ but almost invariant with *k*_*y*_ when the light is *x*-polarized, which means that the image is only differentiated along the *x* direction corresponding to the operator ∂_*x*_^2^. For the same reason, the *y*-polarized input light only induces differentiation along the *y* direction, i.e. ∂_*y*_^2^. In contrast, using circular polarized light makes the transfer function close to *k*_*x*_^2^+*k*_*y*_^2^, corresponding to differentiation along both directions, i.e. ∂_*x*_^2^ + ∂_*y*_^2^. These optical properties of the metasurface enable detection of edges vertical to the polarization of the incident linear polarized light and full edge detection using circular polarized light^31^. We also calculated the transfer functions of the metasurface in the crystalline phase at different polarizations in Fig. 3i–k, which display near invariance to different k-vectors.

**Fig. 4:**
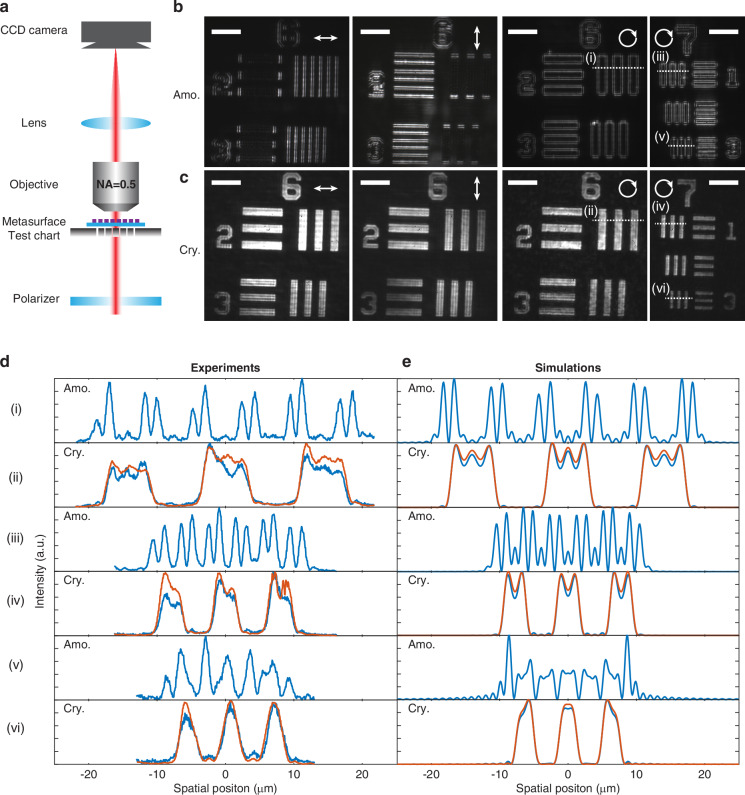
Edge detection and bright field imaging modes. **a** Schematic of the optical imaging setup in experiments, where the metasurface and the test chart are separated by ~0.1 mm. **b**, **c** Optical images of a negative 1951 USAF test target with the existence of the metasurface in the amorphous and crystalline phases. Different linear polarization orientations used in experiments are marked as arrows. Scale bar: 15 μm. **d**, **e** Measured and calculated light intensity profiles of the test target along several dashed lines from (i) to (vi) marked in (**b**) and (**c**). Blue and red curves represent the results with and without the metasurface, respectively. All curves are normalized to the maximum of each

Next, we experimentally demonstrate the phase-change reconfigurability of our nonlocal metasurface, supporting dual imaging functions, i.e., edge detection mode in the amorphous phase and bright field mode in the crystalline phase. The measurement setup is based on a purpose-built microscope schematically illustrated in Fig. 4a. A 1951 USAF negative resolution test target (used here as the imaged object) was illuminated by the same diode laser beam with the wavelength of 1050 nm, and the metasurface was inserted between the test target and the objective to filter the spatial frequencies of the image before being collected by the imaging system. Test target imaging results show the good edge detection capability of the metasurface in the amorphous phase and the selectivity of enhanced edges depending on the incident polarization because of the polarization-sensitive transfer function shown in Fig. 3. The metasurface behaves like a left-right and an up-down edge detection kernel filter with horizontal and vertical polarization orientations, respectively, and full edge detection was implemented by illuminating a circular polarized light (Fig. 4b). To test the resolution of the edge detection operation, we imaged patterns with different group and element numbers on the 1951 USAF test target and observed that the edge detection operation works well for feature sizes larger than 3.5 μm. Compared to the amorphous phase, the measured results from the crystalline Sb_2_Se_3_ metasurface shown in Fig. 4c show straightforward bright field imaging with little differences, other than overall intensity reduction, between the images shown and those without the metasurface (Fig. S9).

In our experiments, we retrieved the gray values as the light intensity along the dashed lines on the captured images in Fig. 4b, c and plotted them in Fig. 4d. We also show in the figure the experimental results obtained with no metasurface present. First, we notice that there is no significant difference in the profile with and without the metasurface in the crystalline phase. The good overlap of the blue and red curves demonstrates the fidelity of the metasurface working in bright field mode, which works well for feature sizes as small as 2.2 μm, the smallest feature size on the test target used in experiments. More imaging results of smaller features are shown in Fig. S10. For the bright field mode, the theoretical resolution limit of our system is 1.05 µm while it is out of the range of the resolution test chart. For the edge detection mode, the width of the obtained edge lines is also limited by the diffraction limitation of 1.05 µm, so it is difficult to clearly distinguish the edges when the feature size is smaller than around 4× diffraction limit (see Fig. S11). When the metasurface operates in the edge detection mode, double lines appear at the edges of patterns due to the introduced Laplacian operator, which coincides well with theoretical results shown in Fig. 4e. In the simulations, we first Fourier transform the real space profile to a k-space profile, then apply the simulated transfer function, and finally inversely Fourier transform it back to real space. Details are described in the Supplementary Information (Note J). Besides, we also quantified the signal noise ratio of our edge detection results, which is >20 (see Fig. S12).

**Fig. 5:**
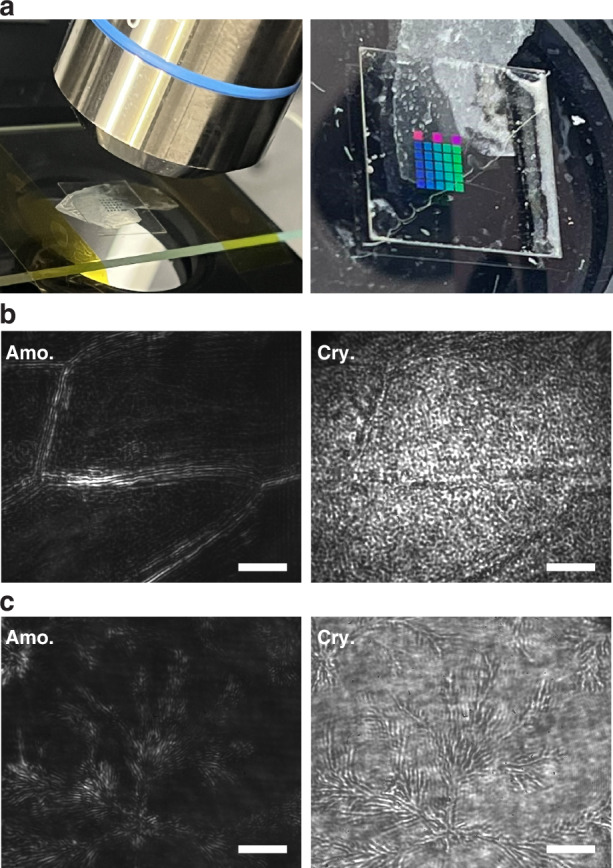
A dual-functionality meta-coverslip for microscopy. **a** A photograph of the phase-change nonlocal metasurface fabricated on a coverslip used to image onion epidermal cells. The different colored blocks are metasurfaces with different periodicities fabricated on the same substrate. **b** The imaging results for onion epidermal cells in the amorphous and crystalline phases of the metasurface. Scale bar: 15 μm. **c** The imaging results for thin sodium carbonate crystals self-grown on glass substrate. Scale bar: 15 μm

Finally, we demonstrate a dual-functionality coverslip based on the phase-change nonlocal metasurface (meta-coverslip), as shown in Fig. 5a. The metasurface was fabricated on one side of an ultrathin coverslip (0.1 mm thick) and the other side of the coverslip was placed in contact with the object to be imaged. The coverslip is made of sapphire, to ensure smaller deformation and less fragility during the fabrication process, but it can readily be replaced by other transparent substrates, such as cheap glass coverslips. The measurement setup and the illumination wavelength are the same as Fig. 4. Figure 5b, c shows the imaging results of onion epidermal cells and sodium carbonate crystals when the metasurface works in the edge detection and the bright field modes. The boundaries and shapes are quite clear when Sb_2_Se_3_ is amorphous, while they become much less identifiable when the metasurface is in the crystalline phase. Although in this work crystallization of Sb_2_Se_3_ is achieved by heating the metasurface on a hot plate at 225°C for 5 min and only one-way switching was shown, we stress that, ultimately, there will be no necessity with the phase-change scheme to insert and remove the metasurface or apply an external force (as in the case of mechanically reconfigurable nonlocal metasurfaces) to switch between the bright field and edge detection microscopy modes. Instead, it is envisaged that in situ switching of the metasurface using integrated microheaters, as demonstrated for example by Zhang et al.^37^, could be implemented and make non-volatile cyclable switching possible.

## Discussion

In conclusion, we have demonstrated a solid-state lossless phase-change nonlocal metasurface working as a reconfigurable spatial frequency filter. The metasurface can perform the dual function of bright field imaging and edge detection imaging. We have experimentally demonstrated one proof-of-concept application, a reconfigurable coverslip, for microscopy using our reconfigurable nonlocal metasurface. Our demonstration opens the door to active nonlocal metasurfaces targeting potential advanced applications such as dynamic spatial frequency filters for different image processing, tunable spacer squeezers^32–34^ and active reciprocal lenses^36^. A parallel work using VO_2_ as the PCM was recently reported^53^. VO_2_ is a volatile PCM, requiring the temperature to be continuously held above threshold to keep the metasurface in one mode. By using a non-volatile PCM, we can eliminate power consumption while the function is being held once the PCM is already set or reset. The state of VO_2_ only depends on the current temperature of the material, but the state of a non-volatile PCM such as Sb_2_Se_3_ is controlled by the past crystallization and amorphization process of the material, which makes dynamic switching non-trivial. Therefore, the next challenge is to address dynamic tunability with integrated heaters to make these metasurfaces switchable at the timescales of the crystallization and the amorphization of these active materials, something that has been achieved in reflective displays^54^, but that may require significant further work for transmissive metasurfaces.

## Methods

### Sample fabrications

The 300 nm thick Sb_2_Se_3_ film was first sputtered on the clean sapphire substrate. Next a 300 nm thick negative tone electron beam resist (ma-N 2403) layer was spin-coated on the Sb_2_Se_3_ film. The pattern was then transferred on the resist layer by EBL (JEOL JBX5500 50 kV with an exposure dose of 250 μC/cm^2^) and the develop process (MF-319 for 1 min then rinsed by DI water). We used the fluorine-based RIE process (Oxford Instruments, CHF_3_, 5 mT, 250 W) to etch the sample and removed the remaining resist by MICROPOSIT Remover 1165 to get the final device. The size of the fabricated metasurfaces is ~400 × 400 μm^2^.

### Optical measurements

The incident angle dependent intensity transmittance of the metasurface at the wavelength of 1050 nm was measured by the same purpose-built setup shown in Fig. 4a, but with a slight modification. The schematic setup is shown in Fig. S13. First, the laser beam from a fiber-coupled diode laser (Thorlabs, MCLS1) was collimated to free space with a beam diameter of ~3 mm, passed through a polarizer, and then slightly focused on the metasurface by a lens (f = 200 mm) to make sure that the spot size is smaller than the area of the metasurface. Then, the transmissive light was collected by an objective with a small NA of 0.1 and coupled to a photodiode power sensor (Thorlabs, S122C). The use of a small NA objective is to make sure the working distance is large enough for sample rotation. The intensity transmittance is calculated by the ratio of the measured power with and without the metasurface.

In this work all optical measurements in the amorphous phase were conducted first after the as-deposited thin film and the metasurface was fabricated. Then we heated the same thin film and metasurface on a hot plate at 225°C for 5 min to fully crystallize the PCM. Finally, we measured all results in the crystalline phase of the metasurface.

## Supplementary information

Supplementary Information for Nonlocal phase-change metaoptics for reconfigurable nonvolatile image processing

## Data Availability

The data that support the findings of this study are available from the corresponding author on request.
