# Supplementary material for: Nonlocal phase-change metaoptics for reconfigurable nonvolatile image processing
Source: Light Sci Appl. 2025 May 6;14:182. doi: 10.1038/s41377-025-01841-x (PMC12053629; doi:10.1038/s41377-025-01841-x)
Supplement: Supplementary file 1 — Supplementary Information for Nonlocal phase-change metaoptics for reconfigurable nonvolatile image processing [file 41377_2025_1841_MOESM1_ESM.docx]

Supplementary Information for

**Nonlocal phase-change metaoptics for reconfigurable nonvolatile image processing**

*Guoce Yang^1^, Mengyun Wang^1^, June Sang Lee^1^, Nikolaos Farmakidis^1^, Joe Shields^2^, Carlota Ruiz de Galarreta^2^, Stuart Kendall^2^, Jacopo Bertolotti^2^, Andriy Moskalenko^1^, Kairan Huang^1^, Andrea Alù^3,4^, C. David Wright^2^*, Harish Bhaskaran^1^**

*^1^Department of Materials, University of Oxford, Parks Road, Oxford, OX1 3PH, UK*

*^2^Centre for Metamaterial Research and Innovation, University of Exeter, Exeter, EX4 4QF, UK*

*^3^Photonics Initiative, Advanced Science Research Center, City University of New York, NY, USA*

*^4^Physics Program, Graduate Center, City University of New York, NY, USA*

**Email:* [*david.wright@exeter.ac.uk*](mailto:david.wright@exeter.ac.uk) and [*harish.bhaskaran@materials.ox.ac.uk*](mailto:harish.bhaskaran@materials.ox.ac.uk)

**A. BIC mode analysis**

We used the Eigenfrequency solver in COMSOL Multiphysics to analyze the eigen modes in this photonic system and identified the BIC mode. The evolution of the mode profile with the varied wave vector is showed in Fig. S1a, confirming that this mode is oriented in the *z*-direction and fully decoupled from free space at *k_x_*=0. The simulations also confirm that the mode becomes more radiative with increased *k_x_*. Next, we quantitively analyze the eigen mode by calculating the eigenfrequency. With an increase of *k_x_*, the real part of the complex eigenfrequency slightly decreases, while the imaginary part shows a large increase (Fig.S1b), mainly contributed by the increased radiative decay rate. This radiative channel transforms the BIC mode from ideal to a quasi-BIC mode, manifested by a lowered Q factor (Fig. S1c).


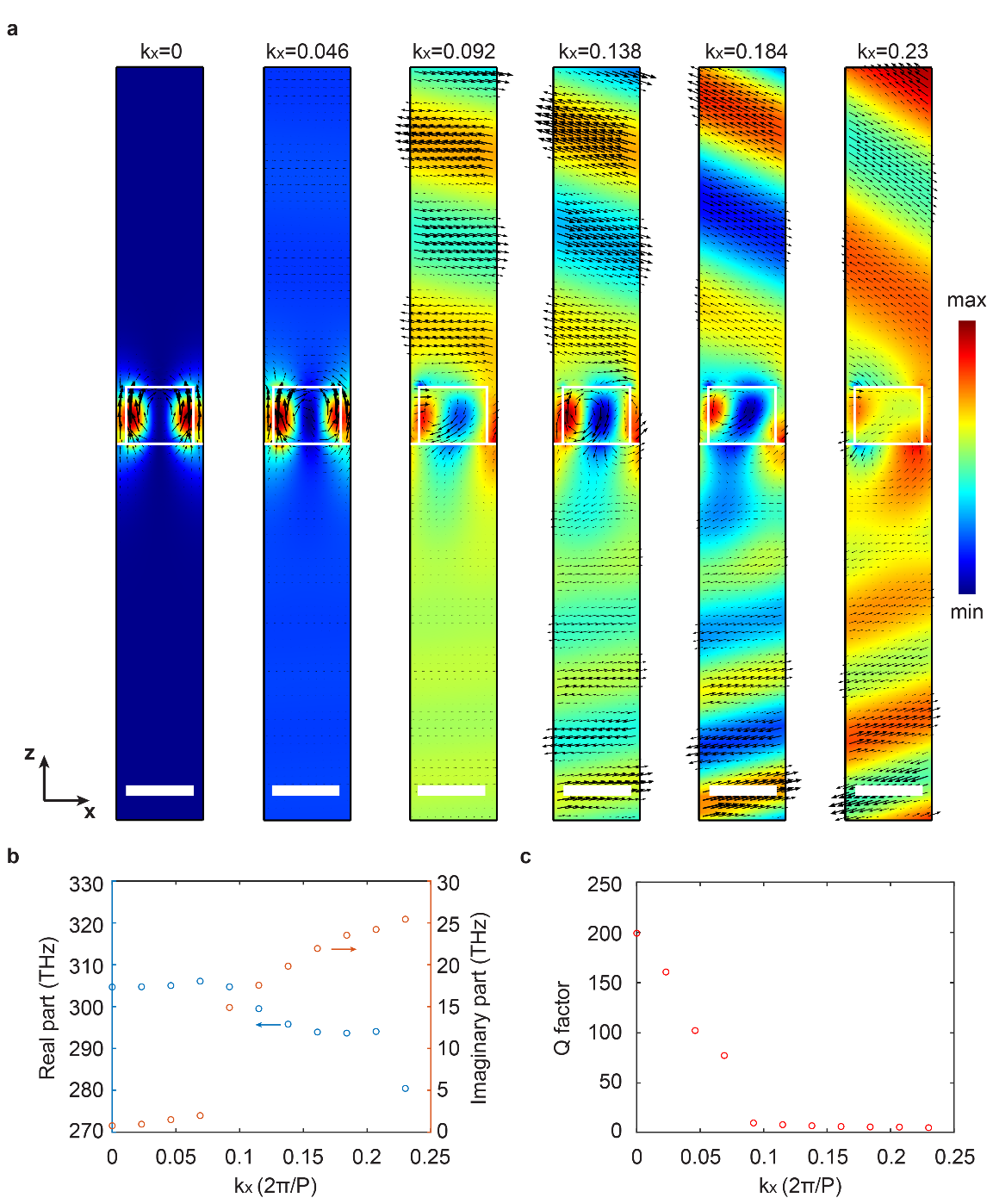


**Figure S1** BIC mode analysis. (a) Simulated eigenmode profiles as a function of *k_x_* (2π/*P*). The length and orientation of arrows represents the magnitude and direction of the electric field, and the color represents *E*_z_ component. Scale bar: 350 nm. (b) Simulated complex eigenfrequency of the q-BIC mode as *k_x_* varies. (c) *k_x_* dependent Q factor of the q-BIC mode.

**B. Comparisons of the metasurface before and after hot plate crystallization**


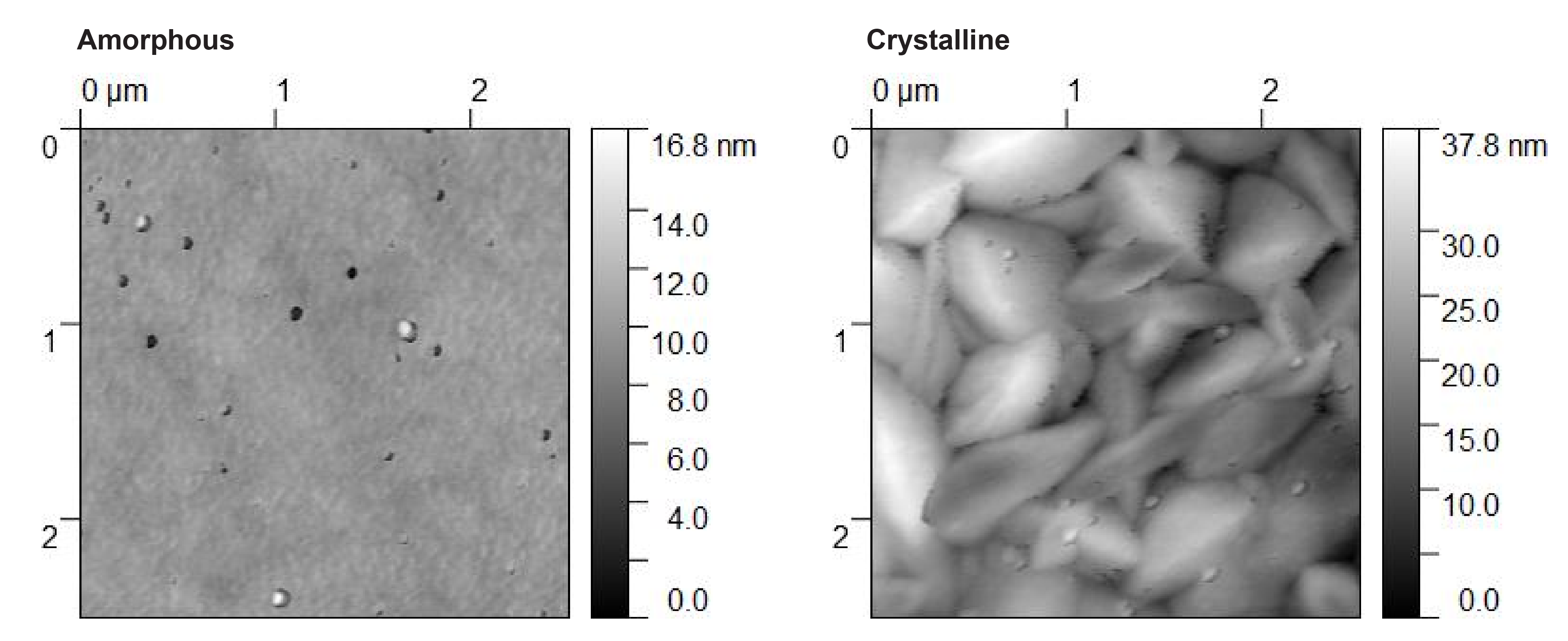


**Figure S2** AFM scanning results of the Sb_2_Se_3_ thin film (283 nm thick) before (Amo.) and after (Cry.) hot plate crystallization. The RMS roughness is 0.69 nm and 6.19 nm before and after crystallization, respective. The thickness experienced 6.7% decrease in average.

**
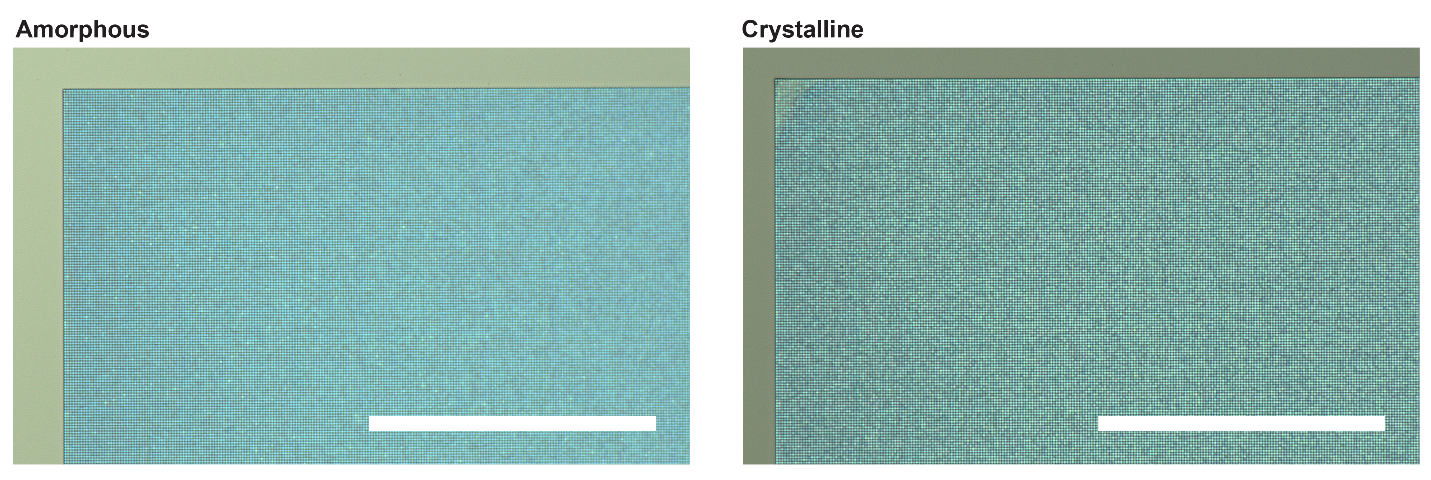
**

**Figure S3** Photographs of the metasurface before and after crystallization by a hot plate. Crystallization makes the metasurface more disordered. Scale bar: 15 μm.


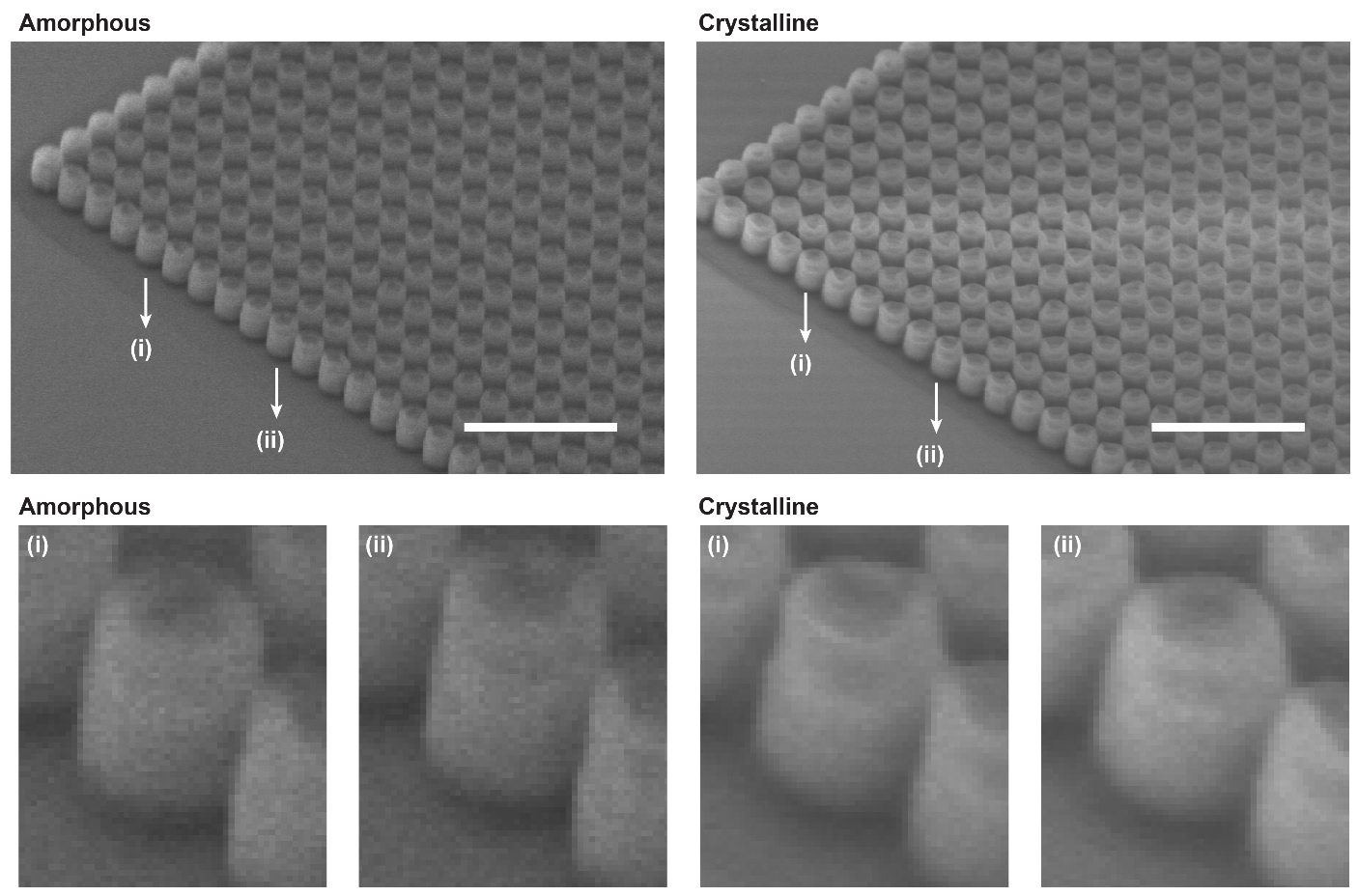


**Figure S4** SEM images of the metasurface before and after crystallization by a hot plate. Slight geometry changes after crystallization can still be observed if we zoom in to compare the individual nanopillars carefully. Scale bar: 2 μm.

**C. Diameter dependent transfer functions**

**
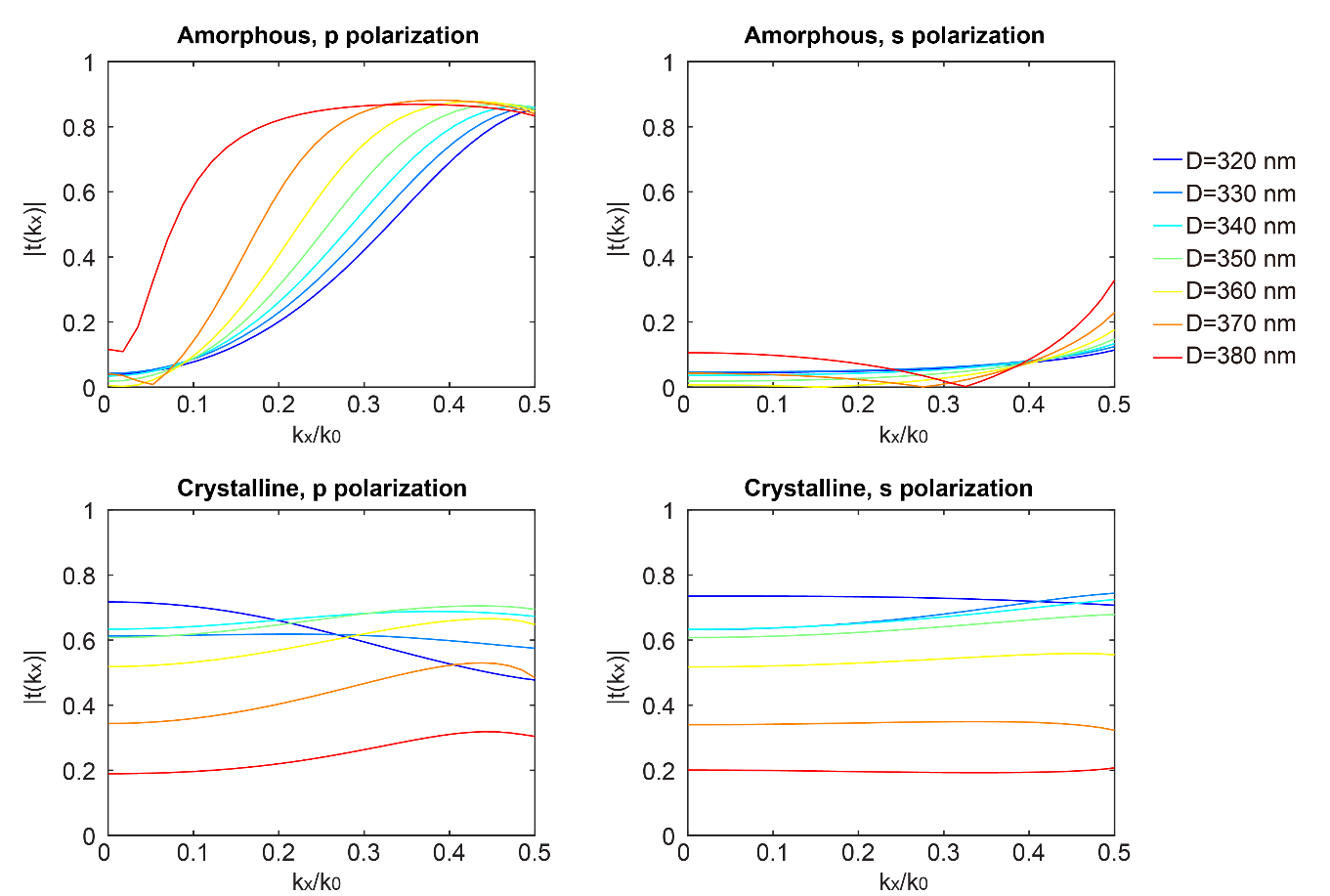
**

**Figure S5** Calculated pillar diameter dependent amplitude of polarized transmittance coefficient in the amorphous and crystalline phases at the wavelength of 1050 nm.

**D. Transfer functions of a disordered metasurface**

To simulate how the nanopillars with random diameters affect the transfer function, we set the super cell including 5×5 unit cells in our simulation model. In each unit cell, the diameter of the nanopillar is a random number obeying uniform distribution centering at 350 nm (shown in Fig. S5a). We changed the variation range of the random number from 0 nm to 40 nm and simulated the transfer function of the metasurface in the amorphous (Fig. S6b) and crystalline (Fig. S6c) phases. We found that the transfer functions still follow the parabolic shape and the maximum value can still reach ~0.9 in the amorphous phase, but in the crystalline phase the efficiency decreases with the increasing variation range of the diameter.


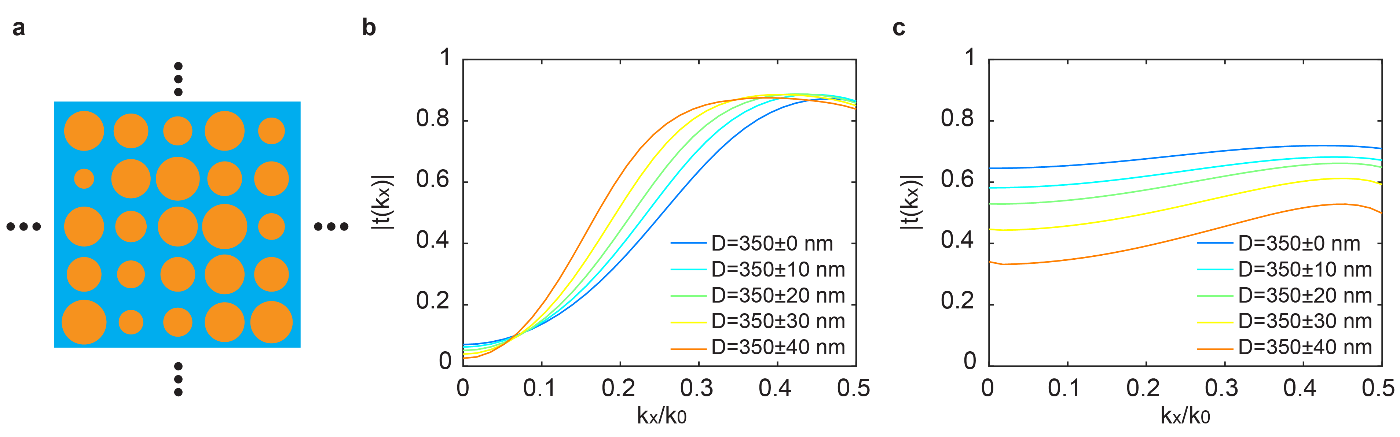


**Figure S6** Transfer functions of a disordered metasurface. (a) Schematic of the model for simulating the disordered metasurface. Note: The relative dimensions of pillars shown in this panel are exaggerated. (b, c) Simulated transfer functions with different diameter variation ranges in the amorphous (b) and crystalline (c) phases.

**E. Cross-polarization transmissive coefficients**

**
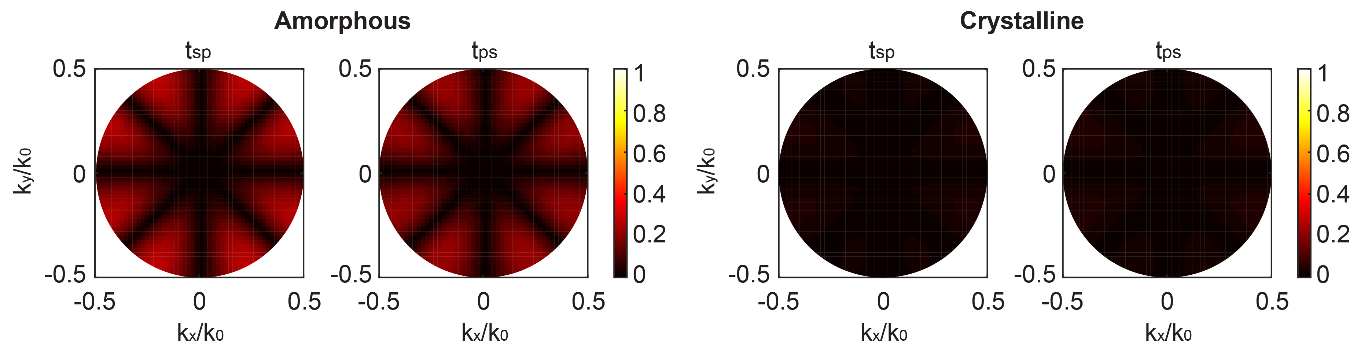
**

**Figure S7** Simulated amplitude of cross-polarization transmissive coefficients of the metasurface in the amorphous and the crystalline phases.

**F. Anisotropic refractive index of the substrate**

The sapphire substrate we used is c-cut, which means the optical axis is perpendicular to the ground plane of the substrate. Here we compare the transfer functions with and without taking account of the anisotropic refractive index of the substrate. In simulations, the anisotropic refractive index tensor of the substrate is $\left[ \begin{matrix} 1.7547 & 0 & 0 \\ 0 & 1.7547 & 0 \\ 0 & 0 & 1.7469 \end{matrix} \right]$, where *z* axis is the optical axis with *n*_o_=1.7547 and *n*_e_=1.7496. The refractive index is set as 1.7547 when the substrate is approximated as isotropic.


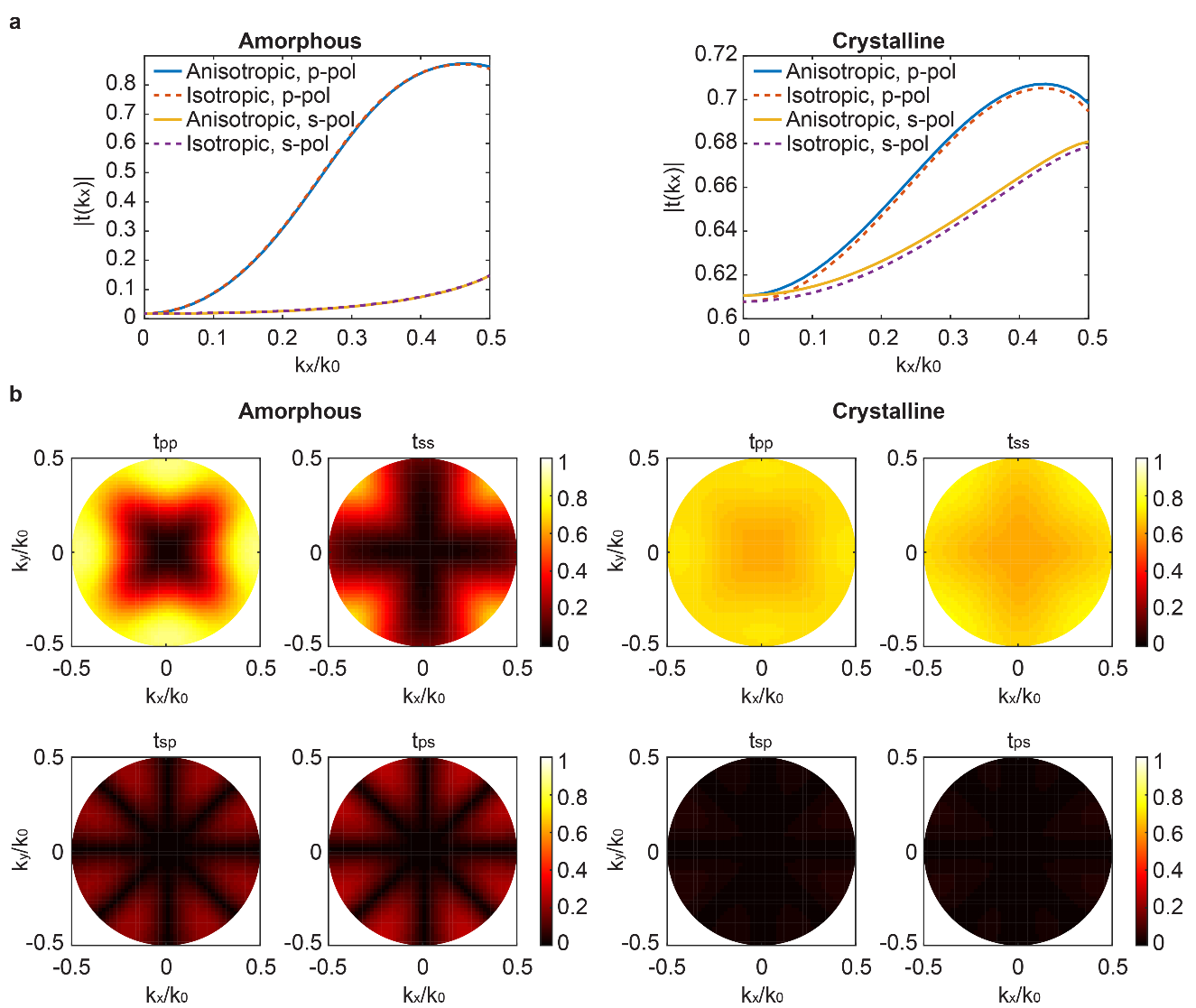


**Figure S8** (a) Comparisons of transfer functions with and without taking the anisotropic refractive index of the substrate into account in amorphous and crystalline phases. (b) Simulated co-polarized and cross-polarized transmissive coefficients as a function of *k_x_* and *k_y_* in amorphous and crystalline phases, which shows no significant differences from results by just treating the substrate isotropic illustrated in Fig. 3b, 3c, 3g, 3h and Fig. S7.

**G. Benchmark of imaging results without the metasurface**


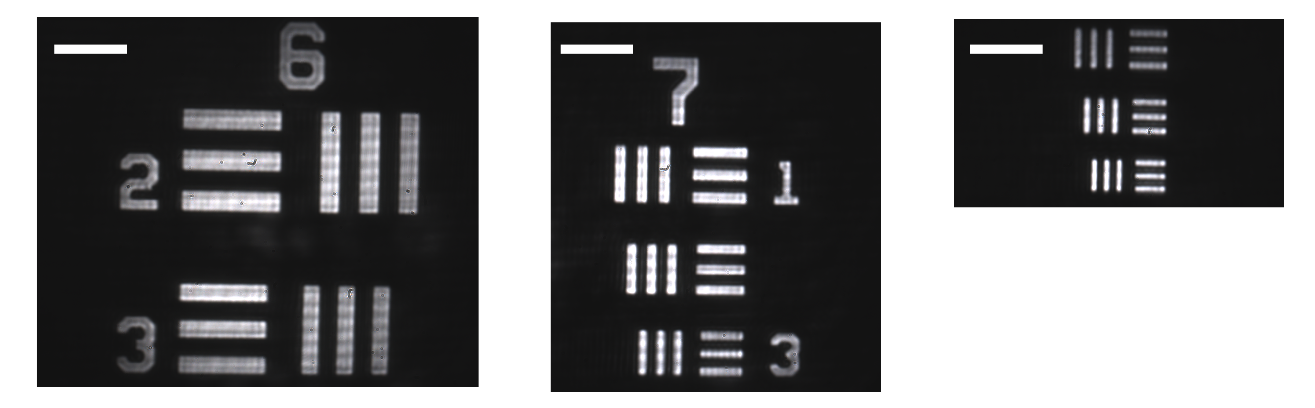


**Figure S9** Imaging results of the test target with no metasurface present. Scale bar: 15 μm

**H. Imaging results of small features with the metasurface**


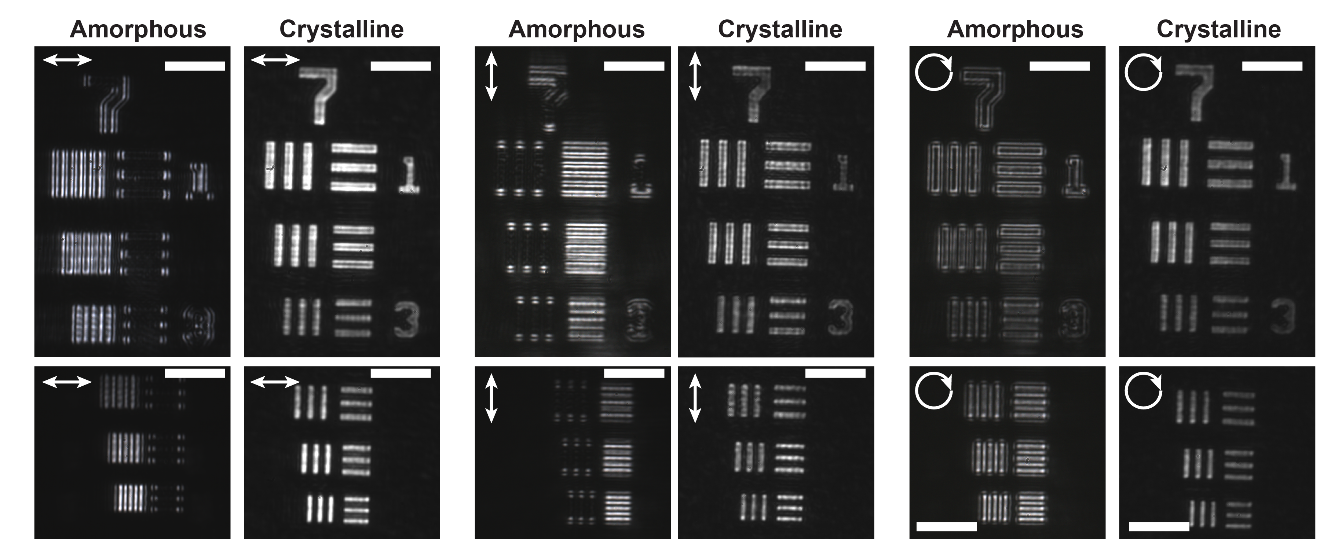


**Figure S10** Imaging results of the test target (Group 7 in the 1951 USAF test chart) with the metasurface present. Scale bar: 15 μm

**I. Resolution analysis**

For the bright field mode, we defined the resolution as the smallest feature size of the line pairs that our optical system can distinguish. Here the smallest feature comes from Group 7 Element 6 on the resolution test chart and the feature size is 2.19 µm. Our system can distinguish the feature very well, which is reasonable because the calculated diffraction limit is only 1.05 µm defined by λ/2NA. Here λ=1050 nm and NA=0.5 in our experiments. For the edge detection mode, we defined the resolution as the smallest feature size of the resolution line pairs that our system can distinguish the edges, which means we should see double peaks at each side of the rectangle function. Finally, we found that the smallest feature that satisfies the standard comes from Group 7 Element 2 corresponding to a feature size of 3.47 µm. In theory, a good edge detection of a rectangle function should have an output with completely separate peaks at both edges (see the illustrative figure below), so the minimum width of the rectangle function should be ~4 times of FWHM corresponding to 4 µm, which also agrees with our reported measured value of 3.5 µm.


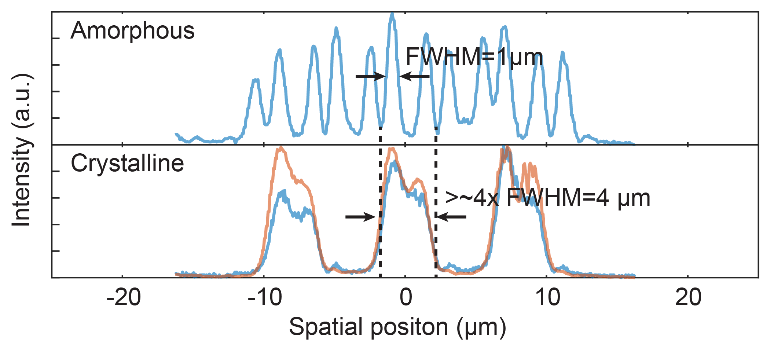


**Figure S11** Illustration of the resolution in the NA limited optical system.

**J. Simulation methods of imaging profiles**

We assume the input light as plane wave with unit amplitude, i.e. *E*_1_(*x*)=1. Then the negative test target pattern (triple lines) works a spatial amplitude modulator with a real-space transmittance function *t*_12_(*x*)=rect[(*x*-2*d*)/*d*]+rect(*x*/*d*)+ rect[(*x*+2*d*)/*d*], where rect(.) is the rectangle function with a unit width and a unit amplitude and *d* is the width of a single line on the test target. The period of the line pair is 2*d*. Then the light immediately modulated by the line pairs is approximately expressed by *E*_2_(*x*)= *E*_1_(*x*) *t*_12_(*x*), and we calculated the field in the spatial frequency space by Fourier transforming *E*_2_(*x*) to *E*_F2_(*k*). We next applied our simulated transfer function of the metasurface *t*_23_(*k*) on it and obtained the light field after the metasurface *E*_F3_(*k*)=*E*_F2_(*k*) *t*_23_(*k*). Finally, the resulted output electric field distribution in real space is calculated by inversely Fourier transforming *E*_F3_(*k*) to *E*_3_(*x*), and the simulated intensity pattern shown the main text is |*E*_3_(*x*)|^2^.

**K. Signal noise ratio of the edge detection**

We define the signal noise ratio (SNR) of the edge detection result as the mean of the signal divided by the standard deviation of noise. Here we show representative measured data of the edge detection results. The signal should be the main peak values of detected edges, which is around 0.25 by average. The noise should be the fluctuation on the signal curve and the calculated standard deviation is 0.0111 (calculated from the interval between -9 µm to -6 µm). Therefore, we estimate the SNR is 0.25/0.0111=22.5.

**
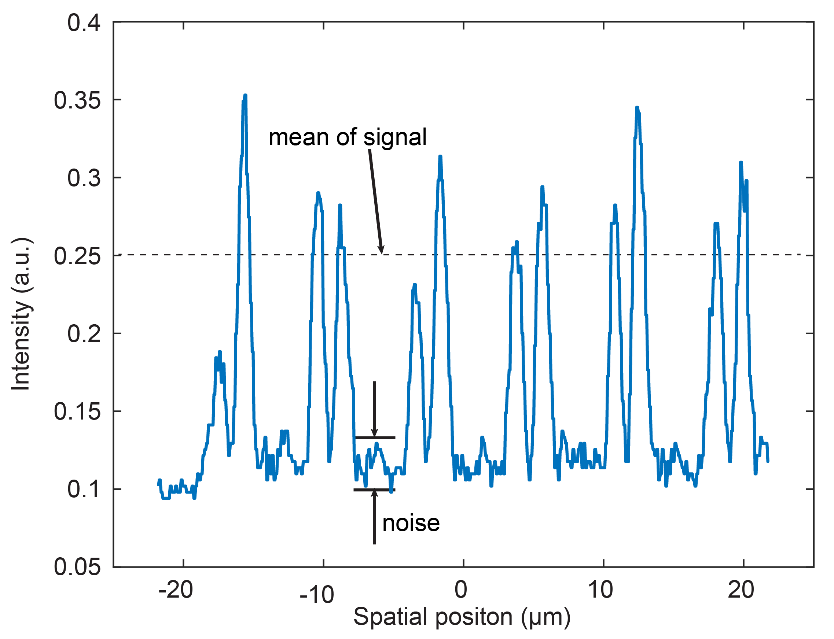
**

**Figure S12** Representative 1D-cut of measured edge detection results.

**L. Optical setup for incident angle dependent transmittance measurements**

**
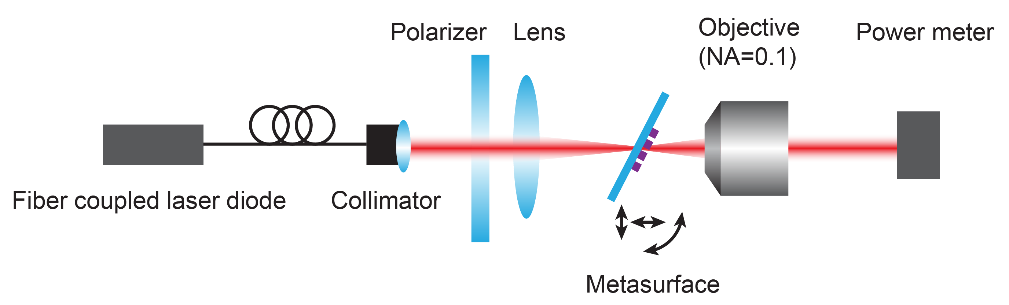
**

**Figure S13** Schematics of optical setup for incident angle dependent transmittance measurements. The metasurface was mounted on a manual 360° rotation stage and the rotation stage was mounted on a manual XYZ translation stage.
